# The association between social media use and dietary intake among adolescents in Türkiye: a cross sectional study

**DOI:** 10.3389/fped.2026.1862690

**Published:** 2026-06-25

**Authors:** Meryem Elif Öztürk

**Affiliations:** Department of Nutrition and Dietetics, Karamanoğlu Mehmetbey University, Karaman, Türkiye

**Keywords:** adolescents, dietary intake, energy intake, social media, sucrose

## Abstract

**Introduction:**

Social media usage has surged up among adolescents and its relationship with dietary intake is unclear. This paper investigated the association between social media use and dietary intake among adolescents in Turkiye.

**Methods:**

A cross sectional study was conducted. Convenience sampling was chosen. Private schools located in two cities in the Central and Eastern regions of Turkiye (Şanlıurfa and Karaman). The sample consisted of 251 adolescents (13–18 years of age) and 49% of them were male. Dietary data were collected using a validated food frequency questionnaire. The multiple regression analysis adjusted for age groups were used to assess the relationship between social media use and dietary intake. Then Benjamini–Hochberg FDR correction was applied. All the data are based on β (unstandardized) with standard error (SE) (β±SE).

**Results:**

Fewer than half of the participants reported using social media for over three hours daily (47.4%). The late adolescents used social media more than three hours a day (*p* < 0.05). Prolonged social media use (>3 h) was associated with higher energy (*β* = 356.21 ± 111.91 kcal, *p* = 0.002), carbohydrate (*β* = 41.19 ± 14.82 g, *p* = 0.006), sucrose (*β* = 10.75 ± 3.24 g, *p* = 0.001), fat (*β* = 16.51 ± 5.83 g, *p* = 0.005), calcium (*β*= 146.44 ± 54.72 mg, *p* = 0.008) and zinc (*β* = 2.16 ± 0.75 mg, *p* = 0.004) intake. Participants who were on social media for more than three hours a day consumed an average of 87.91 g more milk and yoghurt, 27.92 g more meat and processed meat, and 34.38 g more pastry than those who were on social media less than three hours a day. However after Benjamini–Hochberg False Discovery Rate correction, no relationship was found between dairy, meat and pastry consumption with social media duration (*p* > 0.05). Spending more time on social media was also related to more added sugar consumption (*β* = 9.23 ± 3.38 g, *p* = 0.007).

**Conclusions:**

The results indicate that prolonged use of social media is associated with higher energy, fat, sucrose, zinc and calcium consumption. These adolescents have a diet richer in micronutrients but also they follow a more obesogenic dietary pattern, which may be related to an increased risk of inflammatory and chronic diseases later in life. Longitudinal studies are needed to determine a causal relationship.

## Introduction

In the last decade, Turkiye has seen a notable surge in social media platform usage. They have become deeply embedded in daily life, particularly among adolescents who frequently access them through smartphones and tablets. Social media platforms allow adolescents to interact, exchange ideas, forming bonds with peers, and make new friends. However, excessive social media use is associated with mental health problems and physical health issues (1–3).

Beyond their social and communicative functions, social media platforms serve as powerful marketing environments. Children who are subjected to food ads on social media consume more unhealthy foods and beverages (4). Through paid partnerships with influential bloggers admired by adolescents, companies advertise their junk food products on social media (5). As a result, adolescents who spend more time on social media platforms consume more sugar-sweetened beverage and higher-energy dense foods, including sweets and fast foods (6, 7). Moreover, excessive social media use is related to frequently skipping breakfast and consuming fewer vegetables (8). These results indicate that passive exposure to food marketing, brand content, and unhealthy food images on social media is consistently associated with problematic eating behaviors. These findings also suggest that adolescence is a particularly vulnerable period in terms of the adverse effects of social media.

Adolescence is characterized by accelerated physical growth and mental change. It is also a formative period for establishing long-term dietary habits. Adolescents need more nutrition and adopt eating behaviors, which often persist into adulthood. Adolescents in modern societies can access unhealthy dietary options relatively easily (9). Adolescents from middle and high-income families face fewer financial constraints influencing their decisions, which increases their access to unhealthy foods and fast food (9, 10). However, those who develop unhealthy dietary patterns have difficulty meeting their increased micronutrient requirements. Moreover, unhealthy dietary habits contribute to the development of obesity and cardiovascular conditions, diabetes mellitus, and non-alcoholic fatty liver disease later in life. Therefore environmental factors that shape adolescents' eating behavior is of significant public health importance.

Although previous research has explored the relationship between social media use and obesity, the association between social media use and detailed dietary intake among adolescents is unclear. Additionally, adolescents attending private schools who have dominantly middle and high income families may be more influenced by social media due to easier access to a wider variety of foods. Therefore this study sample consists of adolescents attending private schools, which serve as a proxy to reach middle and high-income families. It should be kept in mind that this sample type also imposes limitations in terms of generalization. This study focused on the association between social media use and dietary intake in adolescents attending private schools.

## Material and methods

### Population

**Figure 1:**
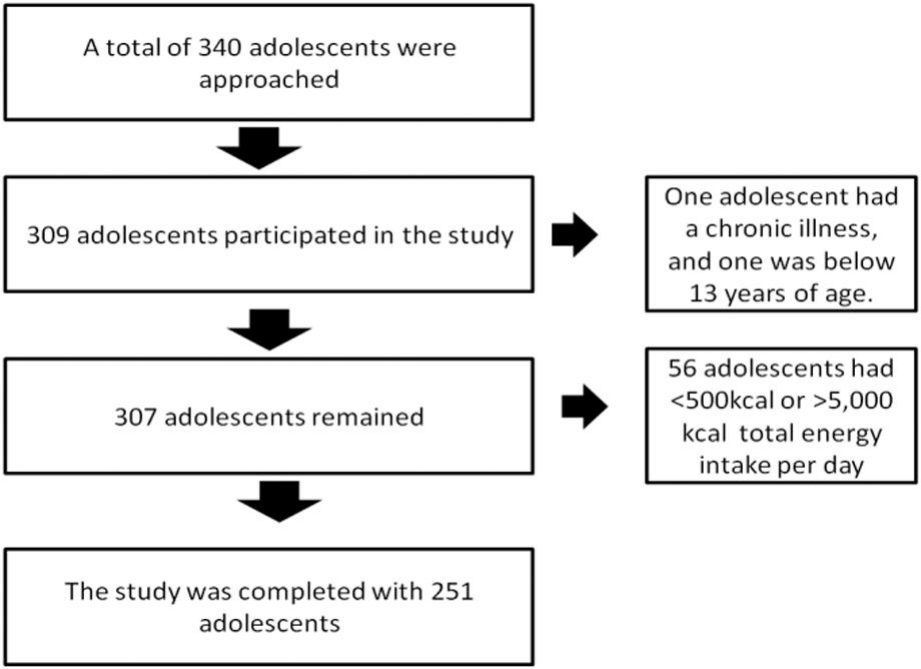
Sample selection flow chart.

The research was conducted between April and June 2023. Based on a study in the literature the sample size was calculated as 240 with a significance level of 0.05% and 90% power (11). The initial sample consisted of 340 adolescents (aged between 13 and 18 years) from private high schools in Şanlıurfa and Karaman in the Central and Eastern regions of Turkey. Convenience sampling was used. The response rate was more than 90%. The exclusion criteria were (1) having a chronic illness, (2) being under 13 years, and (3) having a total energy intake of <500 kcal or >5,000 kcal per day (12). Due to the exclusion criteria the final sample consisted of 251 adolescents. The sample selection flowchart is demonstrated in Figure 1. This study was conducted according to the guidelines laid down in the Declaration of Helsinki. The research received ethical clearance from the Ethics Committee at Karamanoğlu Mehmetbey University (Ethical code: 07-2023/105. Approval date: 05.04.2023). Written consent was received from parents of all participants. Data were collected through face-to-face interviews.

### Data collection

The data were collected using a questionnaire. Age was grouped based on the literature (13). Anthropometric measurements were obtained from school records. A validated Food Frequency Questionnaire (FFQ) was applied to all participants

### Categorization of social media duration

The social media usage was inquired with an open ended question “ How many hours a day do you spend on social media?” Then, the students categorized into two groups (social media use ≤3 h per day; social media use >3 h per day). There are many studies that used single question to detect media use among youths in the literature (11, 14, 15). The cut-off depended on Z scores and previous studies (2, 14).

### Dietary intake

The dietary intake was calculated by a semiquantitative 97-item Food Frequency Questionnaire. The questionnaire has been validated in another study in the literature (16). The food frequencies were “never or rarely,” “once a month,” “twice a month,” “1–2 times per week,” “3–4 times per week,” “5–6 times per week,” “every day,” “every meal.” After gathering the paper-based questionnaires, the researchers proceeded with data entry into the Nutritional Information Systems Package Program (BEBIS 9.0). The food groups were milk and yoghurt, cheese, meat and processed meat, egg, legumes, nuts and seeds, bread, pastry (including bagel, pizza and other pastries), rice, pasta and bulgur, vegetables, potatoes, fruit (inc. fruit juice), added sugar (incl. sugars in sugar sweetened beverages), chocolate, added fats, olives. The average energy and nutrient composition of the consumed foods were also calculated using BEBIS.

### Anthropometric measurements

Anthropometric features were obtained from school records. Anthropometric data were collected during a routine school check-up three months prior to dietary data collection. Body Mass Index (BMI) and BMI for age-Z scores were calculated using weight, height, and WHO AnthroPlus Software (17). Weight status was classified according to World Health Organization (WHO) growth reference standards (underweight: <−2 SD; normal: −2 SD to +1 SD; overweight: >+1 SD to +2 SD; obese: >+2 SD) (18).

### Statistical analysis

Data analysis was conducted using the Statistical Package for the Social Sciences (SPSS) (IBM SPSS Version 21) at a significance level of 0.05. The categorical data were presented as frequencies (percentages). The general and social media use characteristics were analyzed using Chi-square tests. Normality was tested using the Kolmogorov–Smirnov test and Shapiro–Wilk test. The data were analyzed using the student t-test (normal distribution) and the Mann–Whitney U test (nonnormal distribution). Independent samples t-tests were used to compare nutrient intake between social media use groups. The Mann–Whitney U test was employed to identify the relationship between energy and nutrient and food group intake and social media use. The multiple linear regression analysis was performed to examine the association between social media use and dietary intake, adjusting for age groups.

Because the regression analysis comprised 38 dietary outcomes, multiple comparisons were addressed through a two-tier strategy specified a priori. Energy and the three macronutrients (carbohydrate, protein, and fat) were designated as the primary outcomes given their biological proximity to overall dietary exposure, whereas micronutrient and food-group intakes were treated as exploratory secondary outcomes. To control the false discovery rate (FDR) at *q*< 0.05 across the full family of 38 regression outcomes, the Benjamini-Hochberg procedure was applied (19). Adjusted *q*-values are reported alongside raw *p*-values and findings that did not survive FDR correction are interpreted as hypothesis-generating.

## Results

A minority of participants—less than half—indicated daily social media usage exceeding three hours (47.4%).There was no relationship between gender, weight status, and social media use (*p* > 0.05). No difference was observed between genders regarding the relationship between weight status and social media duration (*p* > 0.05) (data was not shown). However, older participants used social media >3 h than their younger counterparts (*p* < .005). Participants who reported spending more than three hours per day on social media were more likely to order food influenced by social media ads (*p* < 0.05). No significant association was found between the type of food ordered and the duration of social media use (*p* > 0.05). Following food-related programs were also not related to the duration of social media use (*p* > 0.05). Adolescents using social media <3 h were more likely to eat faster. However, the difference in social media duration between groups were not statistically significant (*p* > 0.05) (Table 1).

**Table 1:** General characteristics and eating behaviour according to social media use.

Characteristics	Social media use ≤3 h per day (*N* = 132)	Social media use >3 h per day (*N* = 119)	*p*	Effect size
Gender				
Male	60 (48.8)	63 (51.2)	0.236	0.089
Female	72 (56.3)	56 (43.8)		
Age				
13–15 years	53 (62.4)	32 (37.6)	**0**.027*	0.310
16–18 years	79 (47.6)	87 (52.4)		
Weight Status				0.037
Underweight (BMI z score < −2 SD)	2 (66.7)	1 (33.3)	0.926	
Normal weight (BMI z score between −2 SD and 1 SD)	92 (52.6)	83 (47.4)		
Overweight (BMI z score between 1 SD and 2 SD)	30 (53.6)	26 (46.4)		
Obese (BMI z score > 2 SD)	8 (47.1)	9 (52.9)		
Does social media affect your eating behavior?				0.200
Yes	29 (43.3)	38 (56.7)	0.075	
No	103 (56.0)	81 (44.0)		
How often do you place orders based on social media ads?				1.196
Every 2–3 months	53 (62.4)	32 (37.6)	**0**.002*	
Once a month	28 (56)	22 (44)		
2–3 times a month	38 (53.5)	33 (46.5)		
Once a week	10 (45.5)	12 (54.5)		
Few times a week	2 (10)	18 (90)		
Everyday	1 (33.3)	2 (66.7)		
What kind of food do you order based on social media?			0.834	0.054
Fastfoods	85 (53.1)	75 (46.9)		
Home-cooked meals	16 (53.3)	14 (46.7)		
Kebabs	23 (47.9)	25 (52.1)		
Salads	8 (61.5)	5 (38.5)		
Which food-related programs do you follow on social media?				0.123
I do not follow	60 (52.2)	55 (47.8)	0.585	
Healthy eating	32 (60.4)	21 (39.6)		
Recipe videos	25 (48.1)	27 (51.9)		
Dieting	15 (48.4)	16 (51.6)		
Do you use social media while eating?				0.423
Yes	86 (47.5)	95 (52.5)	**0**.010*	
No	46 (65.7)	24 (34.3)		
How does using social media affect your eating style?			0.153	0.332
I eat faster	9 (31)	20 (69)		
I eat slower	51 (54.3)	43 (45.7)		
I eat more	17 (42.5)	23 (57.5)		
I eat less	8 (47.1)	9 (52.9)		

*p* values were obtained using Chi-square test.

Bold indicates *p < 0.05.

Spending more than three hours on social media was related to more daily energy, carbohydrate, protein, fat and sucrose intake (*p* < 0.05). There was no relationship between media use and energy intake from carbohydrate, protein, and fats (%) (*p* > 0.05) (Table 2).

**Table 2:** Association between social media use and energy and nutrient intake.

Energy and nutrients	Social media use ≤3 h per day (*N* = 132)	Social media use >3 h per day (*N* = 119)	*p*	Effect size
	Mean ± SD	Mean ± SD		
Energy	3,591 (1,527)	4,091.8 (1,196.2)	**0**.001*	0.204
Carbohydrate^a^	402.6 ± 112.4	444.4 ± 119.6	**0**.005*	0.361
Carbohydrate % ^a^	46 (10)	48 (9)	0.985	0.001
Sucrose^a^	49 (28.1)	57.5 (30.6)	**0**.002*	0.194
Protein	143.8 ± 41.9	154.1 ± 36.2	**0**.041*	0.262
Protein %^a^	16.5 (3)	16 (3)	0.279	0.069
Fat^a^	144.7 ± 46.23	160.8 ± 51.9	**0**.006*	0.329
Fat %^a^	37 (9)	37 (9)	0.720	0.023
Fiber	35.6 ± 10.2	37.1 ± 9.63	0.210	0.151
Vitamin A^a^	913.3 (973.2)	968.8 (1,090.9)	0.387	0.055
Thiamin	1.99 ± 0.7	2.07 ± 0.6	0.296	0.122
Riboflavin^a^	2.38 (0.9)	2.4 (0.7)	**0**.024*	0.143
Niacin^a^	29.9 (15.4)	31.2 (11.9)	0.177	0.086
Vitamin B6	2.32 ± 0.7	2.46 ± 0.6	0.107	0.214
Vitamin B12^a^	7.32 (4.2)	8.83 (5.5)	**0**.001*	0.203
Vitamin C^a^	119.4 (58.7)	130.1 (67.6)	0.063	0.118
Vitamin E^a^	26.5 (14.6)	29.3 (16.7)	0.067	0.116
Folate^a^	453.1 (188.4)	478 (200.1)	0.367	0.057
Calcium^a^	1,248.5 (590.1)	1,404.1 (450.9)	**0**.005*	0.176
Magnesium	568.9 ± 190.6	614.8 ± 172.3	**0**.047*	0.252
Iron	18.1 ± 5.7	19.5 ± 5.4	**0**.049*	0.252
Zinc	18.3 ± 6.1	20.4 ± 5.6	**0**.006*	0.358

Student T tests were used.

a Median (IQR), Mann–Whitney U test.

Bold indicates *p < 0.05.

More milk and yoghurt intake was related to more social media use (Table 3).

**Table 3:** Association between social media use and food group intake.

Food groups	Social media use ≤3 h per day (*N* = 132)	Social media use >3 h per day (*N* = 119)	*p*	Effect size
	Median (IQR)	Median (IQR)		
Milk and yoghurt	176.5 (212)	253 (314)	**0**.002*	0.194
Cheese	66 (83)	558 (51)	0.190	0.083
Meat and processed meat	166.5 (111)	191 (137)	0.090	0.107
Egg	47 (49)	47 (48)	0.718	0.023
Legumes	15 (18.8)	14 (17)	0.140	0.093
Nuts and seeds	57 (66.8)	59 (80)	0.343	0.059
Bread	236 (174.3)	238 (189)	0.568	0.036
Pastry	146.5 (112.2)	185 (127.9)	**0**.010*	0.163
Rice, pasta and bulgur	47.5 (42.8)	54 (45)	0.454	0.047
Vegetables	151.5 (127)	152 (158)	0.986	0.001
Potatoes	73.5 (45)	75 (67)	0.493	0.043
Fruit	188.5 (173.3)	225 (168)	**0**.039*	0.131
Added sugar	36.5 (30.8)	42 (36)	**0**.011*	0.160
Chocolate	15 (24.5)	20 (32)	**0**.018*	0.149
Added fats	36 (21.75)	40 (23)	**0**.030*	0.138
Olives	10 (19)	10 (14)	0.780	0.018

Mann–Whitney U test.

Bold indicates *p < 0.05.

The multiple regression showed that excessive use of social media was related to more energy (*β* = 356.21 ± 111.91 kcal, *p* = 0.002), carbohydrate (*β* = 41.19 ± 14.82 g, *p* = 0.006), sucrose(*β* = 10.75 ± 3.24 g, *p* = 0.001), protein (*β* = 10.45 ± 5.03 g, *p* *=* 0.039), fat (*β* = 16.51 ± 5.83 g, *p* = 0.005), calcium (*β* = 146.44 ± 54.72 mg, *p* = 0.008) and zinc (*β* = 2.16 ± 0.75 mg, *p* = 0.004) intake. The results showed that the participants who spent more than three hours on social media platforms consumed an average of 87.91 g more milk and yoghurt, 27.92 g more meat and processed meat and 34.38 g more pastry than those using social media less than ≤ 3 h. Longer media use was also related to higher added sugar consumption (*β* = 9.23 ± 3.38 g, *p* = 0.007) and lower legumes consumption (*β* = 4.77 ± 2.29 g, *p* = 0.038). After applying Benjamini–Hochberg FDR correction across all 38 regression outcomes, seven associations remained significant at *q* < 0.05 (sucrose, energy, zinc, fat, carbohydrate, added sugar, and calcium); the remaining associations originally reported as significant under the unadjusted threshold—including milk and yoghurt (*q* = 0.062), pastry (*q* = 0.084), meat and processed meat (*q* = 0.124), legumes (*q* = 0.124), and protein (*q* = 0.124)—did not survive FDR adjustment and are therefore interpreted as hypothesis-generating findings (Table 4).

**Table 4:** Multiple regression analysis of social media use on nutrient and food intake.

Outcomes	β±SE	95% CI	R^2^	*p*	*q* (BH-FDR)
Energy and Nutrients					
Energy	356.21 ± 111.91		0.040	**0**.002*	**0**.**038**^a^
Carbohydrate	41.19 ± 14.82	(11.99–70.39)	0.032	**0**.006*	**0**.**043**^a^
Carbohydrate %	−0.27 ± 0.91	(−2.06–1.52)	0.003	0.766	0.766
Sucrose	10.75 ± 3.24	(4.36–17.14)	0.043	**0**.001*	**0**.**038**^a^
Protein	10.45 ± 5.03	(0.53–20.37)	0.017	**0**.039*	0.124
Protein %	−0.29 ± 0.25	(−0.78–0.19)	0.008	0.230	0.363
Fat	16.51 ± 5.83	(5.03–27.99)	0.031	**0**.005*	**0**.**043**^a^
Fat %	0.64 ± 0.83	(−1.01–2.28)	0.005	0.445	0.532
Fiber	1.41 ± 1.27	(−1.09–3.91)	0.010	0.269	0.393
Vitamin A	177.87 ± 425.07	(−659.33–1,015.07)	0.004	0.676	0.694
Thiamin	0.08 ± 0.08	(−0.08–0.25)	0.005	0.320	0.443
Riboflavin	0.21 ± 0.14	(−0.06–0.48)	0.011	0.123	0.246
Niacin	1.8 ± 1.53	(−1.21–4.81)	0.006	0.239	0.363
Vitamin B6	0.15 ± 0.09	(−0.03–0.32)	0.011	0.102	0.228
Vitamin B12	1.74 ± 1.27	(−0.77–4.24)	0.008	0.174	0.301
Vitamin C	11.04 ± 7.69	(−4.1–26.19)	0.009	0.152	0.289
Vitamin E	2.89 ± 1.53	(−0.14–5.91)	0.014	0.061	0.155
Folate	13.38 ± 21.76	(−29.48–56.24)	0.010	0.539	0.608
Calcium	146.44 ± 54.72	(38.67–254.22)	0.030	**0**.008*	**0**.**043**^a^
Magnesium	44.68 ± 23.29	(−1.2–90.56)	0.016	0.056	0.152
Iron	1.39 ± 0.71	(−0.02–2.79)	0.016	0.053	0.152
Zinc	2.16 ± 0.75	(0.68–3.63)	0.034	**0**.004*	**0**.**043**^a^
Food groups					
Milk and yoghurt	87.91 ± 34.95	(19.08–156.75)	0.026	**0**.013*	0.062
Cheese	−9.5 ± 5.73	(−20.79–1.79)	0.025	0.099	0.228
Meat and processed meat	27.92 ± 13.13	(2.06–53.78)	0.038	**0**.034*	0.124
Egg	−6.51 ± 4.72	(−15.81–2.79)	0.016	0.169	0.301
Legumes	−4.77 ± 2.29	(−9.27–0.26)	0.0,118	**0**.038*	0.124
Nuts and seeds	7.3 ± 7.51	(−7.49–22.09)	0.005	0.332	0.443
Bread	8.35 ± 16.46	(−24.08–40.78)	0.004	0.612	0.646
Pastry	34.38 ± 14.63	(5.56–63.2)	0.022	**0**.020*	0.084
Rice, pasta and bulgur	4.2 ± 4.74	(−5.14–13.55)	0.004	0.376	0.476
Vegetables	9.55 ± 15.73	(−21.43–40.54)	0.003	0.544	0.608
Potatoes	3.04 ± 5.64	(−8.07–14.15)	0.003	0.590	0.641
Fruit	24.22 ± 20.15	(−15.47–63.9)	0.006	0.231	0.363
Added sugar	9.23 ± 3.38	(2.56–15.89)	0.029	**0**.007*	**0**.**043**^a^
Chocolate	4.72 ± 2.93	(−1.06–10.5)	0.010	0.109	0.230
Added fats	3.39 ± 3.53	(−3.57–10.35)	0.006	0.338	0.443
Olives	−0.91 ± 1.19	(−3.25–1.44)	0.003	0.448	0.532

a indicates *q* < 0.05 after Benjamini–Hochberg FDR correction applied across all 38 regression outcomes shown in Table 4 (*k* = 38, *α* = 0.05). BH-FDR, Benjamini–Hochberg false discovery rate.

Multiple regression analysis adjusted for age groups.

Score of 1 indicates social media use > 3 h per day; 0 indicates social media use ≤ 3 h per day.

Bold indicates * *p* < 0.05.

β, beta coefficient; SE, standard error; 95% CI 95%, confidence interval; R2, R squared.

## Discussion

Adolescents are devoting progressively more time to social media use. However, the American Psychological Association (APA) advices that adolescents limit their social media use to maintain both physical and mental health (20). Beyond its psychological effects, advertisements on social media influence eating behavior, significantly. This may be more common among adolescents attending private schools who have dominantly middle and high income families due to their easier access to a wide range of foods. Therefore, this study investigated the association between social media use and energy, nutrient, and food intake in adolescents from high income families.

Age factor is important to understand adolescents' social media behaviors. Social media use was more prevalent among participants between the ages of 16 and 18 than their younger counterparts, which is consistent with the literature (21, 22). As one transitions into young adulthood, one seeks out more social experiences and shows greater interest in interpersonal interactions (21). Thus, older adolescents may be more influenced by social media due to increased exposure.

Regarding eating behaviors, participants with higher social media usage ordered more fast-foods online. The frequent exposure to ads of unhealthy foods may affect their purchase decision (11). In addition, a pattern emerged in which participants who used social media more frequently ate at a faster rate during social media engagement. Bulck and Eggermont (2006) (23) reported that excessive media use was related to eating faster. Adolescents eat faster to use media more comfortably and devote more time to it.

In contrast, weight status was not related to social media use. No difference was observed between genders regarding the relationship between weight status and social media duration, as well. Sampasa-Kanyinga et al. (2015) (24) also reported no relationship between weight status and social media use in adolescents with unhealthy eating habits. On the other hand, male adolescents who spend more than two hours on social media platforms had higher BMI Z scores, in another study (25). The conflicting results might be because of the methodological differences in the evaluation of BMI Z-scores or cultural differences.

Unlike weight status, greater social media use (exceeding three hours daily) was associated with increased intake of energy, carbohydrates, fat, calcium, and zinc. Consistently, the findings revealed a positive association between spending more than three hours on social media and higher intake of milk and yogurt, meat, and pastries. Milk and yogurt are rich sources of calcium, while meat is a significant source of zinc (26). Pastries are energy-dense foods that contain high amounts of carbohydrates (27). These three associations (milk and yoghurt, meat and processed meat, and pastry), however, were attenuated below the *q* < 0.05 threshold after Benjamini–Hochberg correction across the 38 regression outcomes and should therefore be regarded as tentative findings consistent with the dietary pattern outlined above rather than as independently robust associations. Nevertheless, the direction of these associations aligns with the observed nutrient intake profile. Moreover, hamburgers, nuggets, kebabs, and many other fast-food products are generally energy-dense and often contain meat or processed meat (28). Turkish people consume *ayran*—a traditional beverage made from yogurt—alongside kebabs and various fast food dishes. The higher intakes of energy, fat, zinc, and calcium observed among adolescents with greater social media use are therefore compatible with our earlier findings suggesting more frequent consumption of fast foods and kebabs. This conclusion is further supported by previous studies (29, 30). Excessive consumption of fast-food and energy dense foods may cause adolescents to develop colon cancer, cardiovascular diseases, and diabetes later in life (31).

Our findings suggested that sugar intake was associated with prolonged social media use. The sucrose in sugar-sweetened beverages was also included in the “sugars” category. Fawziya et al. (2024) (6) and Khan et al. (2025) (32) found that excessive social media usage is associated with higher consumption of sugar-sweetened beverages and sweets. Social media offers an avenue for food marketers to advertise their products. As social media continues to gain popularity, junk food companies have shifted their marketing efforts to online platforms, targeting young audiences (24). It is known that unhealthy food advertisements are prevalent in social media, which may affect food choices of adolescents (33). Murphy et al. (2020) found that adolescents spend more time looking at advertisements for unhealthy foods compared to advertisements for healthy foods (34). The peer effect may be another reason of high consumption of added sugars, fastfoods and kebabs (35). However, randomized controlled trials showed that adolescents recall unhealthy foods more than healthy posts when coming from celebrities and companies, but not peers (34). Also high energy density snack posts shared by peers do not change desired portion sizes and liking of snacks and sugar sweetened beverages among adolescents (36). Watson et al. (2016) found that adolescents respond faster for high-calorie food images compared to low-calorie food images (37). Images of unhealthy but delicious foods on social media can trigger brain responses related to attention, memory, and reward in adolescents, even after repeated exposure. The appetite state may play a critical role for consumption of unhealthy foods shown on social media. Exposure to unhealthy food images on social media while hungry can lead to unhealthy food choices throughout the day (38). In addition, adolescents having paternal obesity and great appetite for palatable foods are more likely than other adolescents to consume unhealthy and palatable foods, after exposure to their images on social media (39, 40).

Consistent with the literature, our results showed that fruit and vegetable intake was not related to social media use (7). While Khan et al. (2025) (32) reported that prolonged engagement with social media was related to higher fruit and vegetable intake, Byun et al. (2021) (8) found that longer exposure was associated with lower fruit and vegetable intake. The differences in social media exposure across studies may have caused the conflicting results because some researchers defined social media use as exposure to food messages on social media (7), while others defined as leisure-time internet use (8). Cultural differences may be another reason for different results.

This is the first study to investigate the relationship between social media use and detailed dietary intake in adolescents in Turkiye. However, it has many limitations. First, we could not infer causality due to the cross-sectional nature of the research. Second, since the sample was drawn only from two cities and private schools, the generalizability of the study is limited. Third, we did not ask the participants' about their physical activity limits, preventing inferences about weight status. Fourth, the dietary data are based entirely on self-reports and may be subject to recall bias. Fifth social media exposure was measured by adolescents' self-report, which may be subject to desirability bias, recall bias and inaccurate time estimation. As a sixth methodological consideration, the multiple regression analysis examined 38 dietary outcomes simultaneously, which inflates the family-wise Type I error rate. We therefore applied the Benjamini–Hochberg false discovery rate procedure (*q* < 0.05) across all 38 outcomes; associations that did not survive this correction (milk and yoghurt, meat and processed meat, pastry, legumes, and protein) are discussed as hypothesis-generating rather than confirmatory and should be interpreted with appropriate caution. Researchers should conduct further research with larger samples and more detailed questions about social media.

Turkish adolescents from private schools engaging in social media use for over three hours per day consume more energy, carbohydrate, fat, added sugar, zinc and calcium. Excessive energy, fat and sucrose consumption are risk factors for problematic eating. Having and maintaining the problematic eating habits may lead to health issues later in life. Therefore, adolescents should receive nutrition education. Moreover, governments should implement preventive measures against fast food ads on social media. Longitudinal studies are needed to determine causal relationships.

## Data Availability

The raw data supporting the conclusions of this article will be made available by the authors, without undue reservation.

## References

[1] Reid D Weigle P. Social media use among adolescents: benefits and risks. Adolesc Psychiatry. (2014) 4:73–80. 10.2174/221067660402140709115810

[2] Riehm KE Feder KA Tormohlen KN Crum RM Young AS Green KM. Associations between time spent using social media and internalizing and externalizing problems among US youth. JAMA Psychiatry. (2019) 76:1266–73. 10.1001/jamapsychiatry.2019.2325 31509167 PMC6739732

[3] Güneş M Demirer B. The effect of social media use on eating behaviors and physical activity among university students. J Public Health. (2025) 33(2):281–8. 10.1007/s10389-023-02025-w

[4] Mc Carthy CM de Vries R Mackenbach JD. The influence of unhealthy food and beverage marketing through social media and advergaming on diet-related outcomes in children—a systematic review. Obes Rev. (2022) 23(6):e13441. 10.1111/obr.13441 35301815 PMC9286387

[5] Sina E Boakye D Christianson L Ahrens W Hebestreit A. Social media and children's and adolescents’ diets: a systematic review of the underlying social and physiological mechanisms. Adv Nutr. (2022) 13(3):913–37. 10.1093/advances/nmac018 35218190 PMC9156385

[6] Fawziya VR Adi MS Wurjanto M Yuliawati S. Association between the role of peers and social Media exposure with level of sugar-sweetened beverages consumption in adolescents. Amerta Nutr. (2024) 8(3):1–6. 10.20473/amnt.v8i3.2024.383-388

[7] Qutteina Y Hallez L Raedschelders M De Backer C Smits T. Food for teens: how social media is associated with adolescent eating outcomes. Public Health Nutr (2022) 25(2):290–302. 10.1017/S1368980021003116 34325764 PMC8883778

[8] Byun D Kim R Oh H. Leisure-time and study-time internet use and dietary risk factors in Korean adolescents. Am J Clin Nutr. (2021) 114(5):1791–801. 10.1093/ajcn/nqab229 34258617

[9] Majabadi HA Solhi M Montazeri A Shojaeizadeh D Nejat S Khalajabadi Farahani F. Factors influencing fast-food consumption among adolescents in Tehran: a qualitative study. Iran Red Crescent Med J. (2016) 18(3):e23890. 10.5812/ircmj.23890 27247793 PMC4884438

[10] Mumena WA Ateek AA Alamri RK Alobaid SA Alshallali SH Afifi SY. Fast-food consumption, dietary quality, and dietary intake of adolescents in Saudi Arabia. Int J Environ Res Public Health. (2022) 19(22):15083. 10.3390/ijerph192215083 36429802 PMC9690717

[11] Mumena WA Alnezari AI Safar HI Alharbi NS Alahmadi RB Qadhi RI. Media use, dietary intake, and diet quality of adolescents in Saudi Arabia. Pediatr Res. (2023) 94(2):789–95. 10.1038/s41390-023-02505-5 36750738 PMC9903280

[12] Lee MS Carcone AI Ko L Kulik N Ellis DA Naar S. Managing outliers in adolescent food frequency questionnaire data. J Nutr Educ Behav. (2021) 53(1):28–35. 10.1016/j.jneb.2020.08.002 33012663 PMC7855646

[13] Wasiluk A Szczuk J. Underweight, overweight, and obesity in boys and girls at the age of 7–18 years from Eastern Poland in the years 1986–2006. Stud Med (2015) 31(2):99–105. 10.5114/ms.2015.52907

[14] Mougharbel F Chaput JP Sampasa-Kanyinga H Hamilton HA Colman I Leatherdale ST. Heavy social media use and psychological distress among adolescents: the moderating role of sex, age, and parental support. Front Public Health. (2023) 11:1190390. 10.3389/fpubh.2023.1190390 37397708 PMC10310995

[15] Riehm KE Feder KA Tormohlen KN Crum RM Young AS Green KM. Associations between time spent using social media and internalizing and externalizing problems among US youth. JAMA Psychiatry. (2019) 76(12):1266–73. 10.1001/jamapsychiatry.2019.2325 31509167 PMC6739732

[16] Baspinar B Özçelik AÖ. Comparison of commonly used dietary assessment methods in individuals without obesity. Nutr Food Sci. (2021) 51(3):560–77. 10.1108/NFS-05-2020-0192

[17] World Health Organization. WHO AnthroPlus for Personal Computers Manual: Software for Assessing Growth of the world's children and Adolescents. Geneva: WHO (2009) (Available online at: https://www.who.int/tools/growth-reference-datafor-5to19-years). (Accessed March 1, 2026).

[18] World Health Organization. Training Course on Child Growth Assessment. Geneva: WHO (2008).

[19] Benjamini Y Hochberg Y. Controlling the false discovery rate: a practical and powerful approach to multiple testing. J R Stat Soc Ser B. (1995) 57(1):289–300. 10.1111/j.2517-6161.1995.tb02031.x

[20] American Psychological Association (APA) (2023). Health Advisory on Social Media Use in Adolescence. Available online at: https://www.apa.org/topics/social-media-internet/health-advisory-adolescent-social-media-use.pdf (Accessed June 18, 2025)

[21] Politte-Corn M Nick EA Kujawa A. Age-related differences in social media use, online social support, and depressive symptoms in adolescents and emerging adults. Child Adolesc Psychiatry Ment Health. (2023) 28(4):497–503. 10.1111/camh.12640 PMC12051161 36751140

[22] Beeres DT Andersson F Vossen HGM Galanti MR. Social media and mental health among early adolescents in Sweden: a longitudinal study with 2-year follow-up (KUPOL study). J Adoles Health. (2021) 68(5):953–60. 10.1016/j.jadohealth.2020.07.042 32943289

[23] Van den Bulck J Eggermont S. Media use as a reason for meal skipping and fast eating in secondary school children. J Hum Nutr Diet. (2006) 19(2):91–100. 10.1111/j.1365-277X.2006.00683.x 16533371

[24] Sampasa-Kanyinga H Chaput J-P Hamilton HA. Associations between the use of social networking sites and unhealthy eating behaviours and excess body weight in adolescents. Br J Nutr (2015) 114(11):1941–7. 10.1017/S0007114515003566 26400488

[25] Sampasa-Kanyinga H Colman I Goldfield GS Hamilton HA Chaput J-P. Sex differences in the relationship between social media use, short sleep duration, and body mass index among adolescents. Sleep Health. (2020) 6(5):601–8. 10.1016/j.sleh.2020.01.017 32331866

[26] Miller GD Jarvis JK McBean LD. The importance of meeting calcium needs with foods. J Am Coll Nutr (2001) 20(2):168S–85. 10.1080/07315724.2001.10719029 11349940

[27] Olamiti G Ramashia SE. Impact of composite flour on nutritional, bioactive and sensory characteristics of pastry foods: a review. Curr Res Nutr Food Sci. (2024) 12(3):1018–34. 10.12944/CRNFSJ.12.3.4

[28] Bahadoran Z Mirmiran P Azizi F. Fast food pattern and cardiometabolic disorders: a review of current studies. Health Promo Perspect. (2016) 5(4):231. 10.15171/hpp.2015.028 PMC4772793 26933642

[29] Kim HR Han MA. Associations between problematic smartphone use, unhealthy behaviors, and mental health status in Korean adolescents: based on data from the 13th Korea youth risk behavior survey (2017). Psychiatry Investig. (2020) 17(12):1216–25. 10.30773/pi.2020.0007 34724602 PMC8560339

[30] Lwin MO Malik S Ridwan H Au CSS. Media exposure and parental mediation on fast-food consumption among children in metropolitan and suburban Indonesian. Asia Pac J Clin Nutr. (2017) 26(5):899–905. 10.6133/apjcn.122016.04 28802300

[31] Battaglia Richi E Baumer B Conrad B Darioli R Schmid A Keller U. Health risks associated with meat consumption: a review of epidemiological studies. Int J Vitam Nutr Res (2015) 85(1-2):70–8. 10.1024/0300-9831/a000224 26780279

[32] Khan A Feng J Chachay V Tsang JH Huang WY Sit CH. Bytes and bites: social media use and dietary behaviours among adolescents across 41 countries. Pediatr Res. (2025) 98:2101–2108. 10.1038/s41390-025-04030-z 40195543 PMC12811122

[33] Gascoyne C Scully M Wakefield M Morley B. Food and drink marketing on social media and dietary intake in Australian adolescents: findings from a cross-sectional survey. Appetite. (2021) 166:105431. 10.1016/j.appet.2021.105431 34062174

[34] Murphy G Corcoran C Tatlow-Golden M Boyland E Rooney B. See, like, share, remember: adolescents’ responses to unhealthy-, healthy-and non-food advertising in social media. Int J Environ Res Public Health. (2020) 17(7):2181. 10.3390/ijerph17072181 32218252 PMC7177346

[35] Bogl LH Mehlig K Ahrens W Gwozdz W de Henauw S Molnár D, et al. Like me, like you–relative importance of peers and siblings on children's fast food consumption and screen time but not sports club participation depends on age. Int J Behav Nutr Phys Act (2020) 17(1):50. 10.1186/s12966-020-00953-4 32295621 PMC7160987

[36] Sharps MA Hetherington MM Blundell-Birtill P Rolls BJ Evans CE. The effectiveness of a social media intervention for reducing portion sizes in young adults and adolescents. Digit Health. (2019) 5:2055207619878076. 10.1177/2055207619878076 31579525 PMC6757490

[37] Watson P Wiers RW Hommel B Ridderinkhof KR de Wit S. An associative account of how the obesogenic environment biases adolescents’ food choices. Appetite. (2016) 96:560–71. 10.1016/j.appet.2015.10.008 26482282

[38] Sina E Boakye D Christianson L Ahrens W Hebestreit A. Social media and children's and adolescents’ diets: a systematic review of the underlying social and physiological mechanisms. Adv Nutr (2022) 13(3):913–37. 10.1093/advances/nmac018 35218190 PMC9156385

[39] Sadler JR Shearrer GE Papantoni A Yokum ST Stice E Burger KS. Correlates of neural adaptation to food cues and taste: the role of obesity risk factors. Soc Cogn Affect Neurosci. (2023) 18(1):nsab018. 10.1093/scan/nsab018 33681997 PMC10074771

[40] Jensen CD Duraccio KM Carbine KA Barnett KA Kirwan CB. Motivational impact of palatable food correlates with functional brain responses to food images in adolescents. J Pediatr Psychol. (2017) 42(5):578–87. 10.1093/jpepsy/jsw091 27780839

